# Continental estimates of forest cover and forest cover changes in the dry ecosystems of Africa between 1990 and 2000

**DOI:** 10.1111/jbi.12084

**Published:** 2013-03-04

**Authors:** Catherine Bodart, Andreas B Brink, François Donnay, Andrea Lupi, Philippe Mayaux, Frédéric Achard

**Affiliations:** 1 Joint Research Centre of the European Commission, Institute for Environment and SustainabilityIspra, Italy; 2 Reggiani SpA, Joint Research Centre of the European Commission, Institute for Environment and SustainabilityIspra, Italy

**Keywords:** Africa, continental-scale monitoring, deforestation, dry forests, land-cover change, Landsat, uncertainty, woodlands

## Abstract

**Aim:**

This study provides regional estimates of forest cover in dry African ecoregions and the changes in forest cover that occurred there between 1990 and 2000, using a systematic sample of medium-resolution satellite imagery which was processed consistently across the continent.

**Location:**

The study area corresponds to the dry forests and woodlands of Africa between the humid forests and the semi-arid regions. This area covers the Sudanian and Zambezian ecoregions.

**Methods:**

A systematic sample of 1600 Landsat satellite imagery subsets, each 20 km × 20 km in size, were analysed for two reference years: 1990 and 2000. At each sample site and for both years, dense tree cover, open tree cover, other wooded land and other vegetation cover were identified from the analysis of satellite imagery, which comprised multidate segmentation and automatic classification steps followed by visual control by national forestry experts.

**Results:**

Land cover and land-cover changes were estimated at continental and ecoregion scales and compared with existing pan-continental, regional and local studies. The overall accuracy of our land-cover maps was estimated at 87%. Between 1990 and 2000, 3.3 million hectares (Mha) of dense tree cover, 5.8 Mha of open tree cover and 8.9 Mha of other wooded land were lost, with a further 3.9 Mha degraded from dense to open tree cover. These results are substantially lower than the 34 Mha of forest loss reported in the FAO's 2010 Global Forest Resources Assessment for the same period and area.

**Main conclusions:**

Our method generates the first consistent and robust estimates of forest cover and change in dry Africa with known statistical precision at continental and ecoregion scales. These results reduce the uncertainty regarding vegetation cover and its dynamics in these previously poorly studied ecosystems and provide crucial information for both science and environmental policies.

## Introduction

The 2010 Global Forest Resources Assessment (FRA) report published by the Food and Agriculture Organization of the United Nations (FAO, 2010a) estimates the global extent of forests and other wooded land to be 31% of the total land area. At tropical and subtropical latitudes, the percentage of open and closed forest is around 40%, of which 42% is covered by dry forest, 33% by moist forest and 25% by wet and rain forest (Murphy & Lugo, 1986). The world's largest proportion of dry forest ecosystems is in Africa, where they account for 70–80% of forested areas (Murphy & Lugo, 1986). In Africa, this ecosystem is home to more than half of the continent's population – predominantly rural people whose livelihoods depend largely on natural resources (Chidumayo & Gumbo, 2010). The main uses of African dry forests and woodlands are subsistence farming, livestock grazing, timber production and the extraction of fuel wood. The majority of these uses, if not managed sustainably or controlled through appropriate forestry policies and/or management practices, lead to forest degradation and/or destruction. These changes have impacts on the global environment, particularly carbon emissions to the atmosphere and biodiversity loss. These impacts result inevitably in the loss of environmental goods and services, reduced land productivity and threats to human livelihoods. Brink & Eva (2009) noted that the greatest amount of deforestation in Africa is taking place in dry forests, accounting for about 70% of the forest loss between 1975 and 2000 (the humid tropical forests accounted for 16% of the total forest loss in sub-Saharan Africa). Mertz *et al*. (2007) identified African dry forests and woodlands as the most threatened and least protected ecosystem on the continent, largely as a result of population increase, climate change and poor environmental governance and policy frameworks (FAO, 2010b). Moreover, human responses to changing economic opportunities and/or policies (at local, national and global scales) have been highlighted as one of the most important determinants of forest cover change (Geist & Lambin, 2002).

Despite their extensive coverage and importance, Africa's dry tropical forests have received little attention compared to its tropical rain forests, and changes in this ecosystem are still poorly documented at the global scale (Lambin *et al*., 2003). When studying tropical forests, dry forests are frequently either excluded from the study area (Achard *et al*., 2002; Hansen *et al*., 2008) or excluded from the reporting due to high uncertainty (DeFries *et al*., 2002; Ramankutty *et al*., 2007). Recently, Hansen *et al*. (2010) quantified the global gross loss of forest cover between 2000 and 2005 with a combination of coarse-resolution imagery and a stratified sampling of Landsat imagery. The authors estimated that, at the global scale, the dry tropical biome represented 20% of the total forest cover in 2000, with a gross loss of forest cover of 2.9% between 2000 and 2005. However, the status of Africa's dry forests has not been reported. In Africa, FAO national forestry statistics remain the only available source of information (Houghton, 2005). These statistics are collected from national authorities, where available, and inevitably suffer from a lack of consistency and completeness both in time and coverage. They are highly aggregated and of dubious accuracy for some regions (Matthews, 2001; Mayaux *et al*., 2005; Grainger, 2008).

Biome-scale estimates are important to accurately characterize and monitor relative variation within, and potential displacement between, countries in a similar ecological zone (Hansen *et al*., 2008). This information responds to a pressing need for scientific research and support for policy formulation and implementation at national and international levels – in particular the United Nations Framework Convention on Climate Change (UNFCCC) process for the Reduction of Emissions from Deforestation and Forest Degradation (REDD+) and the protection of habitat for biodiversity conservation, as defined in the Convention on Biodiversity (CBD) Aichi Biodiversity Targets (especially Targets 5, 15 and 19).

This study provides estimates of land cover and land-cover change that occurred at the landscape level from 1990 to 2000 in African dry forests and woodlands, with better global consistency and higher accuracy than previously available. Data and processing are based on a systematic grid of Landsat data as part of the global TREES-3 project implemented by the Joint Research Centre of the European Commission to monitor tree cover and its changes across the tropics. The results will be used for the forthcoming FRA 2010 Remote Sensing Survey. The intended outcome of this new survey is to improve the consistency and comparability of forest area and change statistics at regional, ecozone and global levels. Once finalized, results including imagery and land-use classifications at each site will be made available on the FAO FRA web-based portal.

## Materials and methods

### Study area

**Figure 1:**
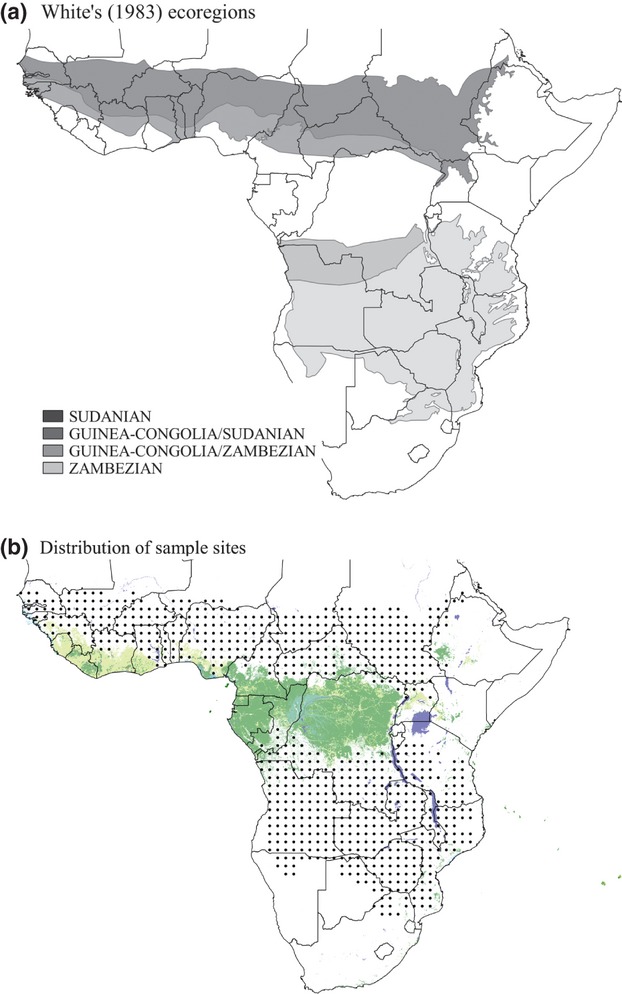
Maps of Africa showing the area of African dry forests and woodlands analysed in this paper according to White's (1983) ecoregions, the distribution of sample sites and excluding the African humid forest domain. (a) Grey-shaded regions correspond to our study area (Sudanian, Guinea–Congolia/Sudanian, Guinea–Congolia/Zambezian and Zambezian ecoregions). (b) A total of 796 sample sites cover the study area. The GLC 2000 evergreen forest classes in the background were used to mask out the sample sites covered by humid forests. Subsets of 20 km × 20 km Landsat images were extracted at each sample site for the reference years 1990 and 2000.

Our study focuses on the dry forests and woodlands of Africa, defined by Chidumayo & Gumbo (2010) as ‘vegetation dominated by woody plants, primarily trees, the canopy of which covers more than 10 per cent of the ground surface, occurring in climates with a dry season of three months or more’. The bioclimatic regions analysed in this paper correspond to the warm subhumid dry forests of the Guinea–Congolia/Sudanian and Guinea–Congolia/Zambezian transition zones and the warm dry woodlands of the Sudanian and Zambezian ecoregions (White, 1983) (Fig. 1a).

In order to consider similar climatic regions, we excluded the semi-arid dry woodlands and grasslands occurring in the Somali–Masai region and the Sahelian and Kalahari–Highveld transition zones. Also excluded are the moist evergreen forests of the Congo basin, the rain forests of West Africa, mangroves, montane and submontane forests and the coastal forest mosaic regions.

The term ‘dry forest’ covers an extensive vegetation type; it includes all the deciduous or seasonal forests between the tropical forests and woodlands to the north and south of the equator. Woodlands range from open woodland to wooded savannas. In West Africa, the subhumid dry forests and dry woodlands correspond to the Guinean and Sudanian savannas. The Guinean subhumid dry forest is characterized by open cover of deciduous trees, but can sometimes form a dense forest. To the north, the Sudanian region is dominated by deciduous shrubland, grass and woody trees interspersed with cropland. In southern Africa, the Guinea–Congolia/Zambezian zone comprises forests or dense woodlands, most of which are highly fragmented by humans or fire. The deciduous miombo woodland (*Brachystegia*–*Julbernardia*) is the most extensive type of vegetation present in the Zambezian region (Chidumayo & Gumbo, 2010). It stretches from Angola almost to the eastern African coast in Mozambique and Tanzania and includes Zambia, southern Democratic Republic of Congo and part of Zimbabwe (White, 1983). In this study, we group the Guinea–Congolia/Sudanian and Sudanian ecoregions under the term Sudanian, and the Guinea–Congolia/Zambezian and Zambezian ecoregions under the term Zambezian.

### Sampling strategy and satellite imagery

Remote-sensing imagery offers the advantages of repetitive data acquisition, a synoptic view of inaccessible areas and consistent image quality over time. Statistical sampling of medium-resolution imagery provides a cost-effective and accurate approach to derive area estimates of land cover and land-cover changes at pan-tropical (Richards *et al*., 2000; FAO, 2001; Achard *et al*., 2002; Hansen *et al*., 2008; Gibbs *et al*., 2010) and continental scales (Brink & Eva, 2009). The sampling design selected for our study consists of a rectilinear grid based on integer degrees of geographical latitude and longitude (Mayaux *et al*., 2005). Subsets of 20 km × 20 km Landsat Thematic Mapper (TM) and Enhanced Thematic Mapper (ETM+) images were used to cover the sample sites for the reference years 1990 and 2000.

A total of 796 sample sites were processed to estimate the areal extent of dry forests and woodlands and the change in each over time. This corresponds to a sampling rate of approximately 3%. Fig. 1b shows the spatial distribution of the selected sample sites. The Sudanian and Zambezian ecoregions are fully covered. For the transition zones within the central Guineo-Congolian ecoregion, the global land-cover map of the African continent (Mayaux *et al*., 2004) for the year 2000 (GLC 2000) was used to mask out the sample sites covered by evergreen forests. The following land-cover classes of the GLC 2000 map were aggregated to represent humid forests: closed evergreen lowland forests; degraded evergreen forests; mangroves; and mosaic forests/croplands. This definition of the African humid forest domain corresponds to the study area of Duveiller *et al*. (2008) over the Congo River basin and the region analysed by Achard *et al*. (2002) of the world's humid tropical forests.

Landsat data were downloaded from the United States Geological Survey's National Centre for Earth Resources Observation and Science (http://glovis.usgs.gov/) at full spatial and spectral resolution (30 m). To find satisfactory imagery in terms of cloud cover and seasonal/radiometric characteristics, Landsat scenes were screened visually and the best available images were selected for each sample site (Beuchle *et al*., 2011). From the total of 796 sites, only 12 sites in Central Africa were excluded from the data set because of poor quality imagery either in 1990 or in 2000. Images taken at the end of the rainy season were given priority because then the discrimination between cropland and natural vegetation is greatest, the cloud coverage is reduced and the impact of fire is limited. The dates of image acquisition were chosen to be as close as possible to the reference years 1990 and 2000. The selected images passed through a preprocessing chain, including the assessment and correction of spatial registration, cloud masking, the conversion to top-of-atmosphere reflectance, haze correction and normalization. More details about these preprocessing steps and their impacts on the results can be found in Bodart *et al*. (2011).

### Data processing

The processing approach combines multidate segmentation, supervised classification and visual checks and refinement of class labels by forestry experts. The full classification and change-detection approach is described in Raši *et al*. (2011). Object-based classification was performed to produce land-cover maps at each sample site. Labels of final objects (minimum mapping unit of 3 ha) were based on the proportional area of the different land-cover categories contained in these objects. Classes were assigned according to the following aggregation rules:

- Dense tree cover (≥ 70% tree cover portion in segment)
- Open tree cover (30–70% tree cover portion)
- Other wooded land (≥ 70% shrubs, forest regrowth)
- Other land cover (including croplands, herbaceous cover and bare land)
- Inland water

The ‘tree cover’ portion is based on the FAO forest definition criteria for canopy density (≥ 10%) and tree height (≥ 5 m). Natural forests and forest plantations are included in the ‘tree cover’ class, as is tree cover outside forests, such as in parks or on agricultural land. The ‘open tree cover’ class includes both a mosaic of dense tree cover (patches of dense forest with cleared areas) and open tree cover fragmented by small crop fields (< 3 ha) or highly degraded by wood harvesting or burning. The term ‘other wooded land’ is used for any woody vegetation layer less than 5 m high, which includes mainly shrubland, but also shrub-like agricultural crops (such as coffee and tea), vegetation regrowth or plantations with small trees. ‘Other land cover’ groups together all non-woody vegetation land covers (e.g. herbaceous cover, pastures, non-woody crops, bare soils and settlements), with the exception of ‘inland water’. Clouds and their shadows were masked during the preprocessing steps.

Statistics on forest cover and changes in forest cover between the two dates were extracted from the multidate objects. In order to generate the same sampling probability for each site and account for the variation in acquisition date, three successive correction steps were applied to account for: (1) linear adjustment of change matrices to reference dates; (2) replacement of missing data; and (3) weighting of sample sites, based on the curvature of the Earth, for handling unequal sampling intensity. Details and formulae can be found in Eva *et al*. (2012) and Appendix S1 of the Supporting Information.

For the study area, the total land-cover area can be extrapolated from the average proportion using the Horvitz–Thompson direct expansion estimator (see Särndal *et al*., 1992, for a general discussion of Horvitz–Thompson estimators). The application and utility of the direct expansion method has been used in various studies (Brink & Eva, 2009; Eva *et al*., 2010). The total class area *Z*_*c*_ is obtained from 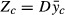, where *D* is the total area of the study region and 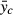 is the average proportion of land cover for a particular class *c*.

The estimation of the variance of the mean was based on a local estimation of the variance (Eva *et al*., 2012). The annual change rates were calculated by dividing the total area of change by the time period and by the average total area of cover over the two dates.

The sampling approach (distribution, sample and unit size) has been designed to provide statistically valid (i.e. with a relatively small standard error) estimates at continental to regional scales (Bodart *et al*., 2011). Statistics were therefore reported only at the continental and ecoregion levels.

### Accuracy assessment

A thorough assessment of accuracy would require independent reference data which could be considered more reliable and accurate than the data set to be assessed. In the current case, very high-resolution data or field observations would provide appropriate information to assess the accuracy of maps derived from Landsat imagery (Strahler *et al*., 2006). However, no such reference data exist for Africa, especially for the years 1990 and 2000. Therefore, an accuracy analysis with a more limited scope was conducted through an independent analysis of a subsample of 338 randomly selected Landsat subsets (out of the total of 2043 sample units for Africa). For each of the 338 primary sample units, five points were systematically selected within the 20 km × 20 km subset. The objects falling on these 338 × 5 points were re-interpreted carefully by an independent expert at two dates (1990 and 2000) to create an independent data set for assessing the ‘consistency’ of the land-cover class labels. The systematic selection resulted in 1552 labelled objects (a few sample units are missing). As a large majority of these systematically selected objects did not show any change in land cover between 1990 and 2000, we also selected all the changed objects (i.e. objects showing changes according to our interpretation) falling on a denser systematic grid of 9 × 9 points with 1 km spacing for assessing the accuracy of changed objects. A total of 1194 changed objects were selected in this manner (from a population of 25,688 systematic points) and interpreted by the independent expert together and non-differentially with the 1552 objects.

When analysing the 1552 systematic polygons used for land-cover accuracy, there is an overall agreement of 87% for the six land-cover classes and 94% for the tree-cover classes. If we were to use these points to estimate areas, the relative difference between areas from our interpretation and from the independent assessment would be 7.9%. When comparing our change results to the independent assessment of 1552 systematic points complemented by a second set of 1194 ‘change’ points, the agreements for the changes between the tree cover classes and other classes are 97.5% and 90.2% for the two sets of points, respectively. In addition, 7.2% of the objects mapped as unchanged were considered to have changed by the independent expert, and conversely, 17.3% of the changed objects were unchanged according to the independent assessment.

IDL codes have been used for the random selection of the Landsat subsets and the systematic selection of objects inside each subset. The same tool was used for the interpretation of the selected objects as that used for the visual checking of classification results (Raši *et al*., 2011). Statistics of change were extracted from the objects and confusion matrices were produced in Excel.

## Results

### Status of dry forests and woodlands

**Figure 2:**
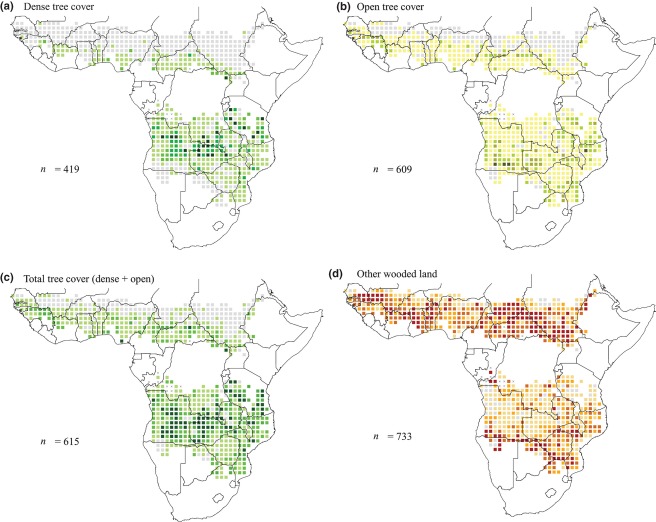
Maps of Africa showing the areal proportions of tree cover and other wooded land cover for the year 2000 in African dry forests and woodlands. At each sample site, land cover was identified from analysis of 20 km × 20 km Landsat image subsets. (a) Dense tree cover; (b) open tree cover; (c) total tree cover (dense and open); (d) other wooded land. Four categories from pale to dark represent areal proportions of [0.001, 0.25], (0.25, 0.5], (0.5, 0.75] and (0.75, 1], respectively; the remaining sites with a land-cover proportion < 0.001 are in light grey. Crosses correspond to missing data; *n* = total number of sample sites where the land cover has been identified.

Our results show that in the year 2000, the study area was covered by 263.3 million hectares (Mha) of tree cover, of which 118.2 Mha were dense tree cover and 145.1 Mha were open tree cover (Table 1). Other wooded land covered 444.8 Mha. The areal extent of dense and open tree cover was 37% of the entire Zambezian ecoregion and 11% of the Sudanian ecoregion. For the study area, the Zambezian ecoregion accounted for 87% and 70% of the total area in dense and open tree cover, respectively (Table 1, Fig. 2). Other wooded land covered 31% of the Zambezian ecoregion and 50% of the Sudanian ecoregion.

**Table 1:** Estimates of tree cover and other wooded land cover for the year 2000 for the whole dry African region and the two sub-ecoregions (in 10^6^ ha and percentage of the total area, both given ± standard error). The distribution of land cover (%) per ecoregion is given in italics. Inland water and other land cover have not been reported

	Whole region	Sudanian	Zambezian
Dense tree cover	118.2 ( ± 6.5)	15.1 (± 2.8)	103.1 (± 5.9)
	10.8% ( ± 0.6)	2.8% (± 0.5)	18.8% (± 1.1)
	*100%*	*13%*	*87%*
Open tree cover	145.1 ( ± 5.0)	44.1 (± 2.9)	101.0 (± 4.1)
	13.3% ( ± 0.5)	8.1% (± 0.5)	18.4% (± 0.8)
	*100%*	*30%*	*70%*
Total tree cover (dense + open)	263.3	59.2	204.1
	24.1%	10.9%	37.2%
	*100%*	*22%*	*78%*
Wooded land	444.8 ( ± 9.2)	273.0 (± 6.8)	171.8 (± 6.2)
	40.7% ( ± 0.8)	50.2% (± 1.3)	31.2% (± 1.1)
	*100%*	*61%*	*39%*
Total study area	1093	543.2	549.8
	100%	100%	100%

### Changes in forested area

We estimate that between 1990 and 2000, 3.6 Mha of dense tree cover was converted to other wooded land or other land cover, and 8.7 Mha of open tree cover and 18.7 Mha of other wooded land were converted to other land cover (Table 2). During the same period, the region gained 0.3 Mha of dense tree cover, 2.9 Mha of open tree cover and 9.8 Mha of other wooded land, leading to a total net loss of 3.3 Mha of dense tree cover, 5.8 Mha of open tree cover and 8.9 Mha of other wooded land. In addition, net changes from dense to open tree cover totalled 3.9 Mha. The annual net rates of loss of dense and open tree cover were estimated at 0.28% and 0.39%, respectively, while 0.32% of the study area was, on average, converted annually from dense to open tree cover. The annual net rate of other wooded land loss was estimated at 0.20%.

**Table 2:** Estimates of changes in land cover in the dry forests and woodlands of Africa from 1990 to 2000 (in 10^6^ ha, ± standard error). Inland waters are not reported

1990/2000	Dense TC*	Open TC*	OWL*	OLC*	Total, 1990
Dense TC	116.9 ± 6.5	4.9 ± 0.4	1.6 ± 0.3	2.0 ± 0.3	125.4 ± 6.7
Open TC	1.0 ± 0.1	137.3 ± 4.9	5.6 ± 0.4	3.1 ± 0.3	147.1 ± 5.0
OWL	0.2 ± 0.1	2.4 ± 0.2	427.6 ± 9.1	18.7 ± 0.9	449.2 ± 9.3
OLC	0.1 ± 0.0	0.5 ± 0.1	9.8 ± 0.5	345.3 ± 9.3	356.7 ± 11.9
Total, 2000	118.2 ± 6.5	145.1 ± 5.0	444.8 ± 9.2	369.6 ± 11.9	1093

* TC = tree cover; OWL = other wooded land; OLC = other land cover.

The net loss of total tree cover (dense and open) was estimated to be 9.1 Mha. This total tree cover was converted to other wooded land and other land cover in almost the same proportions as the net changes (4.6 Mha and 4.5 Mha, respectively). The gross gain to dense and open tree cover, however, occurred mainly from other wooded land, especially in the open tree cover, where conversion from other wooded land accounted for 83% of the total increase from 1990 to 2000 (2.4 Mha vs. 0.5 Mha from other land cover) (Table 2).

**Figure 3:**
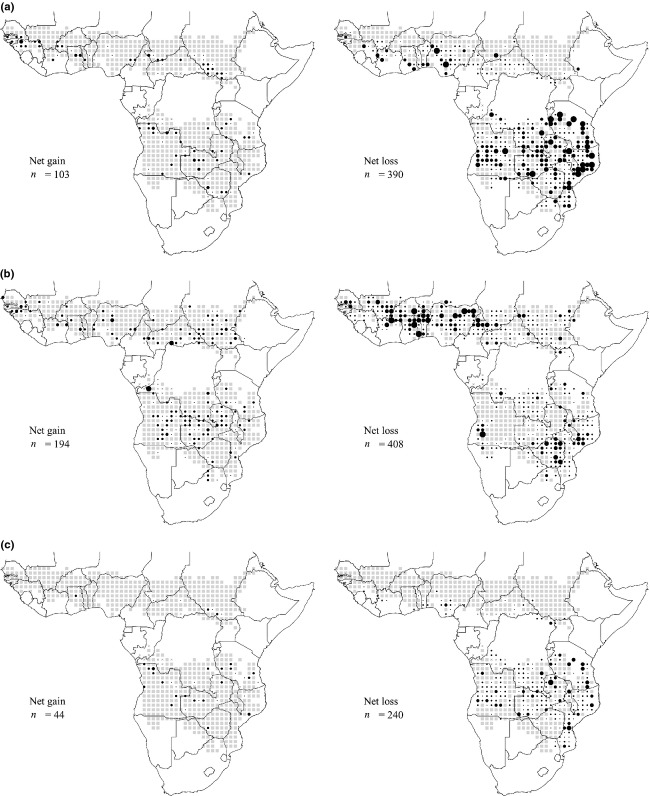
Maps of Africa showing the net changes in tree cover and other wooded land from 1990 to 2000 in African dry forests and woodlands. At each sample site and for both years, land cover was identified from the analysis of 20 km × 20 km Landsat image subsets. (a) Net change from tree cover (dense and open) to other wooded land and other land cover; (b) net change from other wooded land to other land cover; (c) net change from dense to open tree cover. Net gain on the left and net loss on the right; circle size indicates change in area in seven categories: [0.1, 1]; (1, 10]; (10, 20]; (20, 30]; (30, 40]; (40, 50]; and > 50 km^2^; the remaining sites with change area < 0.1 km^2^ are in light grey, and crosses correspond to missing data; *n* = number of sample sites where the land-cover change has been identified.

Analysis of the distribution of changes in forest cover by ecoregion shows that the Zambezian region accounts for more than 80% of the total change in tree cover (Table 3, Fig. 3). In this ecoregion, 7.6 Mha of total tree cover was lost between 1990 and 2000 (annual net rate of 0.37%), predominantly from open tree cover, while the Sudanian ecoregion lost 1.5 Mha of total tree cover (annual net rate of 0.25%). Net changes from dense to open tree cover follow the same pattern, with 3.6 Mha in the Southern Hemisphere and 0.3 Mha in the Northern Hemisphere. In terms of other wooded land, the Sudanian ecoregion represents almost 70% of the total area lost (net and gross). In this ecoregion, 5.9 Mha was converted from other wooded land to other land cover, while 3 Mha was lost in the Zambezian ecoregion.

**Table 3:** Estimates of gross loss and gain of forest cover and net change (in 10^6^ ha, ± standard error) from 1990 to 2000 for the whole dry African region and the two sub-ecoregions. The annual rates of change are given in italics (total area of change divided by the time period and by the average total area of cover over the two dates)

		Whole region		Sudanian		Zambezian	
Total tree cover (dense + open)	Gross loss	−12.3 ± 0.9	*0.46%*	−2.9 ± 0.5	*0.48%*	−9.4 ± 0.7	*0.45%*
	Gross gain	3.2 ± 0.3	*0.12%*	1.4 ± 0.2	*0.23%*	1.8 ± 0.2	*0.09%*
	Net change	−9.1	*0.34%*	−1.5	*0.25%*	−7.6	*0.37%*
Other wooded land	Gross loss	−18.7 ± 0.9	*0.42%*	−12.8 ± 0.8	*0.46%*	−5.9 ± 0.5	*0.34%*
	Gross gain	9.8 ± 0.5	*0.22%*	6.9 ± 0.5	*0.25%*	2.9 ± 0.3	*0.17%*
	Net change	−8.9	*0.20%*	−5.9	*0.21%*	−3.0	*0.18%*
Dense to open tree cover	Gross loss	−4.9 ± 0.4	*0.40%*	−0.5 ± 0.1	*0.32%*	−4.4 ± 0.4	*0.41%*
	Gross gain	1.0 ± 0.1	*0.08%*	0.2 ± 0.1	*0.13%*	0.8 ± 0.1	*0.08%*
	Net change	−3.9	*0.32%*	−0.3	*0.19%*	−3.6	*0.34%*

**Figure 4:**
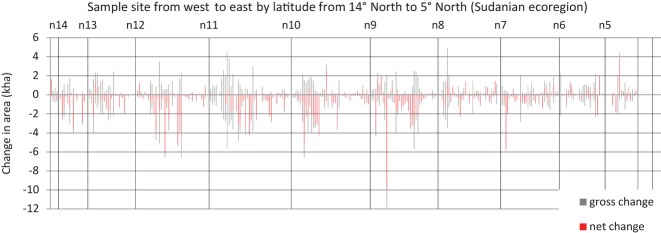
Gains and losses between other wooded land and other land cover identified at each sample site in the Sudanian region showing the intra-site variability. Land conversion in both directions (loss and gain), from and to other wooded land can be observed inside the same sample site. Gross and net change area are represented (negative change corresponds to a conversion from other wooded land to other land cover; sample sites with no change are not shown).

High dynamism in both gains and losses between other wooded land and other land cover has been observed in the same proportion in both ecoregions. Over this period of 10 years, about half of the total area of other wooded land converted to other vegetation cover has been offset by gains from other vegetation cover to other wooded land. Moreover, 75% of the sample sites where changes in other wooded land cover were identified exhibited land conversion in both directions (loss and gain), from and to other land cover. In some sample sites, this intra-site variability results in a very small or zero net change (Fig. 4 gives examples for the Sudanian ecoregion).

## Discussion

### Comparison at continental level

For the first time, changes in land cover in the African dry ecosystems were estimated at the continental level using a consistent processing of medium spatial resolution data. To the best of our knowledge, no previous study has provided comparable results at this level of accuracy and consistency. According to our sampling of Landsat observations, the Sudanian and Zambezian ecoregions of Africa are covered by about 700 Mha of dry forests and woodlands (263 and 445 Mha, respectively), i.e. approximately 20% of the continental land is covered by this biome. Compared to the GLC 2000 map for the same study area, this result is slightly superior to the total area covered by deciduous forests, dry woodlands and shrublands land-cover categories (659 Mha), and corresponds to three times the area covered by the African humid dense forest (235 Mha). Our distribution maps and statistics by ecoregion show that the Zambezian ecoregion contains the largest areal extent of dry forests, mainly the deciduous miombo type, which is especially dense in Angola, Zambia, the southern Democratic Republic of Congo, Tanzania and Mozambique. In the Sudanian ecoregion, the remaining dry forests are essentially open forests and are much more sparsely distributed, with some concentrations in Guinea, Nigeria, Cameroon and the Central African Republic. However, the majority of woodlands and shrublands are found in this ecoregion, often densely aggregated, for example in southern Senegal and Mali, northern Ghana and Benin, the Central African Republic, Chad and Sudan. [This study was done before Sudan had officially split into Sudan and South Sudan.]

The total area of land-cover change has been estimated from and to dense tree cover, open tree cover and other wooded land. Between 1990 and 2000, the annual net losses of tree cover and other wooded land were estimated at 0.91 Mha and 0.89 Mha, respectively, with an additional 0.39 Mha converted from dense to open tree cover. Based on the most recent national forestry reports (FAO, 2010a) for the same set of 30 countries and excluding the humid domain, the annual net area of deforestation between 1990 and 2000 is estimated at 3.4 Mha (see Appendix S2 for more detail). This total loss of forest area is significantly higher than our estimates for tree cover and other wooded land losses. The difference is even bigger when compared to the country data reported in the FRA 2000 report (FAO, 2001) for the same time period (see Appendix S2 for a comparison of the two reports). In this older report, the annual net forest loss between 1990 and 2000 was estimated at 4.2 Mha. Sudan and Zambia alone accounted for 0.9 and 0.8 Mha, respectively. In the FRA 2010, these two countries revised their estimates downward to 0.6 and 0.3 Mha annual net forest loss, respectively, while Tanzania and Mozambique increased them to 0.4 and 0.2 Mha, respectively. Côte d'Ivoire changed from a net loss of 1.2 Mha (FAO, 2001) to a net gain of 50 Mha between 1990 and 2000 in the last FRA report (FAO, 2010a). In terms of total forest cover in 1990, the estimates have been revised upward from 498 Mha in the FRA 2000 to 542 Mha in the FRA 2010. These discrepancies highlight, once again, the pressing need for better estimates in these regions and emphasize the advantage of our harmonized methodology for forest monitoring in the dry Africa biome.

Compared to the humid biome, our estimates of forest loss (0.34% annual net loss of total tree cover and 0.32% annual net loss of dense to open tree cover) are significantly higher than the rates estimated by Duveiller *et al*. (2008) for Central Africa (0.16% and 0.09%, respectively). The method used by Duveiller *et al*. (2008) is comparable to the one used in this study in terms of time period (1990–2000), image processing (object-based classification) and sampling approach (systematic sampling every 0.5 degrees). However, their study area did not cover the humid forests of West Africa. For the humid tropical forests of Africa, covering the whole Guineo-Congolian zone and Madagascar, Achard *et al*. (2002) estimated the annual net deforested area between 1990 and 1997 at 0.71 Mha, plus a further 0.39 Mha of degradation, leading to annual average net rates of 0.36% and 0.21%, respectively. The authors also emphasized the very high local rates found in Madagascar and Côte d'Ivoire. This study was based on visual interpretation of 19 subsets of Landsat scenes located over ‘hotspots’ of deforestation in the humid tropical forest.

### Comparison at regional and local level

According to our study, 84% of the total deforested area occurred in the Zambezian ecoregion. This amount rises to 92% when considering the net loss from dense to open tree cover. Large spatially concentrated areas of loss can be found in Mozambique, Tanzania, Zambia and Angola, while isolated hotspots are present in Nigeria and the border of the humid forest in Ghana.

Several local studies based on Landsat data over the same time period have identified decreases in forests and woodlands belonging to the miombo ecosystem in southern Africa – for example, Mozambique (Jansen *et al*., 2008), Angola (Cabral *et al*., 2011) and Malawi (Palamuleni *et al*., 2011), where results indicate annual local deforestation rates generally higher than 2%. Agricultural expansion was highlighted as the main driver of deforestation, although fuel wood extraction (firewood and charcoal for urban areas) is also contributing considerably to forest degradation and deforestation, especially when the wood is cut by a ‘clear-felling system’ (Chidumayo & Gumbo, 2010) and the sites are consequently converted to croplands. These local studies usually concentrate on landscapes with fast changes or those under threat, which can explain the higher rates of change measured in these areas.

In terms of deforestation in wooded land, the Sudanian ecoregion accounted for 67% of the total gross loss. These changes correspond mainly to changes in land use from and to agriculture. Four intensive zones of change can be distinguished: (1) in north-eastern Nigeria and Cameroon; (2) in northern Benin, Togo and Ghana; (3) in southern Mali and Burkina Faso; and (4) in southern Senegal. While no change could be detected in some areas, we noted that some sites were already entirely covered by agriculture in 1990. This is particularly the case in southern Niger, north-western Nigeria and central and northern Burkina Faso. Considerable research has been conducted over the Sudano-Sahelian region since the 1960s (Anyamba & Tucker, 2005; Olsson *et al*., 2005). These studies used data at much lower resolution and have shown that the region has experienced an increase in vegetation greenness over recent decades. Fensholt *et al*. (2012) demonstrates that this greening trend of the Sudano-Sahelian region is primarily driven by precipitation and that higher precipitation values occurred mainly during the summer months of the observed period 2002–2007, compared to the 1980s. However, even if highlighting a coupled increase in precipitation and normalized difference vegetation index (NDVI), these low-resolution-based studies fail to provide more detailed information on the qualitative changes in vegetation structure and cover. Very few local studies exist where detailed vegetation dynamics are monitored based on medium-resolution imagery in these regions. Some examples can be found in Ghana (Pabi, 2007) and Mali (Tappan & McGahuey, 2007). Our results show an annual net loss of other wooded land of 0.6 Mha (or 0.21% annual loss) in the Sudanian ecoregion, but also reveal a very dynamic landscape with changes, both positive and negative, occurring in most cases within the same sample site. This high variability reflects the agricultural system of shifting cultivation employed by the majority of farmers in West Africa.

In West Africa in particular, a major phase of conversion of natural vegetation such as forests and other wooded land to agriculture occurred before the 1990s. Brink & Eva (2009) and Gibbs *et al*. (2010) – both Landsat-based studies – document increases in agricultural areas of nearly 60%, largely at the expense of forests, over sub-Saharan Africa, beginning in the mid 1970s, with the greatest agricultural expansion occurring in West Africa. However, with current global policies and investments such as the growing bioenergy market (Amigun *et al*., 2011), the increased incidence of ‘land-grabbing’ (Cotula *et al*., 2009) and the ‘leakage’ or displacement of deforestation to other countries (Meyfroidt *et al*., 2010), this ‘slowing’ deforestation trend could again accelerate due to new land-resource needs. Continued observations of forests are required to monitor and assess the status of, changes to and pressures on this valuable resource.

### Limits of digital classification and data

If the dry forest areas of Africa are poorly studied, it is not only due to a lower level of interest in these regions but also because of the considerable difficulties in mapping seasonal forests and detecting changes. Mapping vegetation changes in these ecosystems is a challenging task and the risk of mis-estimation is high. The same type of deciduous vegetation shows very different spectral response during the dry leafless season and the (green) wet season. Therefore, images of the same land cover acquired during different seasons can lead to two different automatic land-cover classifications although there is no change in land cover. During the data selection, pairs of images from the same month were given preference in order to reduce overestimation of change due to different vegetation phenology. Nevertheless, imagery at a similar seasonal stage could not be found for over 30% of the data set. The difference in acquisition date and interannual variability resulted in relatively poor-quality outputs coming from the automatic post-classification change detection. Visual interpretation by regional experts was therefore a mandatory and relatively prolonged process in accurately mapping forest cover and changes in forest cover in these ecosystems. Efforts have been made to visually check every polygon of vegetation change using a consistent and harmonized interpretation across countries and over time. In order to reduce the time needed to achieve a similar quality, a more advanced methodology could be investigated, including textural analysis or automatic multitemporal analysis of biannual imagery complemented by the use of annual MODIS (Moderate Resolution Imaging Spectroradiometer) data time series to identify appropriate acquisition dates within the year.

Landsat data allow for the detection of most changes occurring at the landscape level, but the spatial resolution does not permit the assessment of differences in species composition or fine-scale degradation of canopy cover. Moreover, for a cost-effective approach, we decided not to map changes smaller than 3 ha. Our study considered two classes of tree cover (dense and open), based on the proportion of tree cover inside of an object. The ‘open tree cover’ class could be composed of patches of dense tree cover with other land cover in between or it could correspond to tree cover fragmented by small crop fields or highly degraded by wood harvesting or burning. The transition between dense and open tree cover can therefore be interpreted as forest degradation or deforestation, depending on the definition used for these processes.

## Conclusions

African dry forests and woodlands are characterized by relatively poor forest protection and unsustainable management practices caused largely by insufficient knowledge of resources. In addition, the increasing human population with a strong dependence on the environmental services provided by forests represents one of the major challenges to forests and the forestry sector, particularly in the drylands of sub-Saharan Africa (FAO, 2010b). For the first time, a detailed and consistent analysis of changes in land cover has been conducted over the whole African dry region using a global sampling of Landsat imagery and a region-specific harmonized approach. The robust methodology adopted reduced considerably the uncertainties in vegetation cover and its dynamics and enables consistent monitoring over time and space. Between 1990 and 2000, the study indicates that 3.3 Mha of dense tree cover, 5.8 Mha of open tree cover and 8.9 Mha of other wooded land were lost, with a further 3.9 Mha degraded from dense to open tree cover. These results are substantially lower than the 34 Mha previously reported by the national authorities in the FRA 2010 over the same study area (FAO, 2010a). However, the large surface area of African dry forests and woodlands (three times the area covered by the African humid dense forest), the high vulnerability of millions of people living in and around the forests and the high annual rate of deforestation (0.34%) lend a global significance to the loss of vegetation cover in these ecosystems. This study provides accurate estimates of deforestation and degradation rates at continental and ecoregion levels, and a global picture of hotspots of deforestation. These results will enable improved formulation and effective monitoring of international environmental policies. In particular, such data provide crucial information to accurately estimate forest carbon fluxes and assess the potential opportunity/impact of REDD+ mitigation activities and other sustainable forest management policies in African dry forests and woodlands. The results open the way to more research quantifying the impact of biodiversity conservation law and the achievements set in the Aichi Targets as defined in the CBD at the global scale, or locally by intensifying the number of samples inside and outside protected areas for detailed assessments. New threatened areas can be identified by overlaying our results on the loss of natural vegetation and hence habitat loss with maps of species occurrence and abundance or biodiversity value. Better input on land-cover change will improve land-cover modelling and enhance our understanding of the causes and impact of land-cover change processes at continental scales. By highlighting the magnitude and intensity of changes that occurred in dry African forests and in view of their potential socio-economic and environmental impacts, this study reinforces the need to direct more attention and resources to this threatened but previously poorly studied ecosystem.

## Biosketch

**Catherine Bodart** worked for three years at the Institute for Environment and Sustainability of the Joint Research Centre (JRC), contributing to the TREES-3 Project. She is pursuing a PhD in Environmental Engineering at the Earth and Life Institute of the Université Catholique de Louvain (Belgium). Her scientific interests deal with forest monitoring, satellite-image processing and dry forest ecosystems. She is currently working as an international consultant in the Forestry Department of the Food and Agriculture Organization of the United Nations. The Institute for Environment and Sustainability is one of the seven institutes of the European Commission's Joint Research Centre (JRC). It provides scientific analysis on the sustainable management of natural resources at the European and global level, with a particular expertise on land-cover change in the tropics (http://ies.jrc.ec.europa.eu/).

Author contributions: C.B., P.M. and F.A conceived the ideas and developed the methodology; C.B., A.B., F.D. and A.L. collected the data; C.B., A.B. and P.M. analysed the data; C.B., A.B. and P.M. led the writing.

## SUPPORTING INFORMATION

**Appendix S1** Statistical methods.

**Appendix S2** Comparison of FAO national statistics.
