# Supplementary material for: Continental estimates of forest cover and forest cover changes in the dry ecosystems of Africa between 1990 and 2000
Source: J Biogeogr. 2013 Mar 4;40(6):1036–47. doi: 10.1111/jbi.12084 (PMC3736226; doi:10.1111/jbi.12084)
Supplement: Supplementary file 1 [file jbi0040-1036-SD1.docx]

**Supporting Information**

**Continental estimates of forest cover and forest cover changes in the dry ecosystems of Africa between 1990 and 2000**

Catherine Bodart, Andreas B. Brink, François Donnay, Andrea Lupi, Philippe Mayaux and Frédéric Achard

*Journal of Biogeography*

**Appendix S1** Statistical methods

Statistics on forest cover and changes in forest cover between the two dates (1990 and 2000) were extracted from the multidate objects. In order to generate the same sampling probability for each site and respect the reference dates, three successive correction steps were applied to account for (1) linear adjustment of change matrices to reference dates, (2) replacement of missing data, and (3) weighting of sample sites based on the curvature of the Earth, to control for unequal sampling intensity. These correction steps are described below. More details can be found in Eva *et al.* (2012).

**1.** Images representing the year 1990 were acquired at different dates around 1990 (in the range 1984–1994). Images representing the year 2000 were collected in the years 1998–2003. To account for this variation in acquisition date, the land-cover change matrices were linearly adjusted to the reference dates 30 June 1990 and 30 June 2000. Rates of change in land cover were assumed to be constant over the given period (Eva *et al*., 2010).

**2.** For the missing sample sites, we used a local average from surrounding sample sites as surrogate results. The following weights (δ*_jj_*_′_) were applied for the local average of land-cover change matrices of surrounding sample sites:

where *d*(*j*,*j*′) is the distance between two sites.

**3.** Sample sites are taken at each one-degree intersection of longitude and latitude. Due to the curvature of the Earth, the sampling probability increases with the latitude and leads to higher intensity sampling in higher latitudes. In order to correct for such unequal sampling probability, sample sites were given a weight equal to the cosine of the latitude for producing the land-cover change matrix over the whole region. The weighted average of proportion per class is then given by the following formula:

$\bar{y_{c}}=\frac{\sum_{i=1}^{n} y_{ic}\cos\theta_{i}}{\sum_{i=1}^{n} \cos\theta_{i}}$,

where *n* is the number of sample units, *y_ic_* is the proportion of land cover for a particular class *c* in the *i*-th sample unit and θ*_i_* is the latitude of the centre of the *i*-th sample site.

Cloudy areas were considered an unbiased loss of data, and assumed to have the same proportions of land cover as non-cloudy areas within the same site. This is achieved by converting the land-cover change matrices to area proportions relative to the total cloud-free land area of the sample site.

The usual variance estimation of the mean is known to have a positive bias (Stehman *et al.*, 2011). Alternative estimators based on a local estimation of the variance have been shown to reduce the bias (Wolter, 1984). We used an estimator of the standard error based on local variance estimation:

$s^{2}=\left(1-f\right)\frac{\sum_{j\neq j'} w_{jj'}\delta_{jj'}\left(y_{j}-y_{j'}\right)^{2}}{2\sum_{j\neq j'} w_{jj'}\delta_{jj'}}$,

where *f* is the sampling rate, the weight *w_jj′_* is an average of the weights *w_j_* and *w_j′_* and δ*_jj_*_′_ is a decreasing function of the distance between *j* and *j′* (note that if we choose δ*_jj_*_′_ = 1, *j* ≠ *j′*, we have the usual variance estimator). The standard error (s.e.) is then calculated as:

$se=\frac{s}{\sqrt{n}}$,

where *n* is the total number of available sample sites.

**References**

Eva, H.D., Carboni, S., Achard, F., Stach, N., Durieux, L., Faure, J.-F. & Mollicone, D. (2010) Monitoring forest areas from continental to territorial levels using a sample of medium spatial resolution satellite imagery. *ISPRS Journal of Photogrammetry and Remote Sensing*, **65,** 191–197.

Eva, H.D., Achard, F., Beuchle, R., de Miranda, E., Carboni, S., Seliger, R., Vollmar, M., Holler, W.A., Oshiro, O.T., Barrena Arroyo, V. & Gallego, J. (2012) Forest cover changes in tropical South and Central America from 1990 to 2005 and related carbon emissions and removals. *Remote Sensing*, 4, 1369–1391.

Stehman, S.V., Hansen, M.C., Broich, M. & Potapov, P.V. (2011) Adapting a global stratified random sample for regional estimation of forest cover change derived from satellite imagery. *Remote Sensing of Environment*, **115,** 650–658.

Wolter, K.M. (1984) An investigation of some estimators of variance for systematic sampling. *Journal of the American Statistical Association*, **79,** 781–790.

**Appendix S2** Comparison of FAO national statistics

Figure S1 compares the country data (net loss between 1990 and 2000 and total forest cover in 1990) reported in the FAO’s Global Forest Resources Assessment (FRA) 2000 (FAO, 2001) with the same data reported 10 years later in the FAO FRA 2010 (FAO, 2010) for the same time period. The FAO statistics were not reported by forest type. For instance, the annual net changes in area for the Democratic Republic of Congo, the Central African Republic, Cameroon and Côte d’Ivoire do not distinguish between humid evergreen forest and dry forest. To correct this effect, we multiplied the national area reported in the FAO FRA reports by the proportion of dry forest in the country as identified in the global land-cover map of the African continent (Mayaux *et al.*, 2004) for the year 2000 (GLC 2000), thus excluding the humid domain. By doing so, the annual net area of forest cover loss over the 30 African countries analysed in the study between 1990 and 2000 reported by the FAO FRA 2010 is reduced from 3.9 to 3.4 Mha.

The FAO national statistics concern land-use classes and land-use changes, therefore deforestation is reported only if the forest area has been converted to other land uses. In a forest reserve, an area under regrowth conditions might appear as other wooded land in our land-cover classification while still a forest in terms of land use, i.e. the total area of land use change should be less than total area of land-cover change.

**Figure S1** Total area reported in the FAO FRA 2000 and reviewed in the FAO FRA 2010 for the 30 African countries analysed in the study. The area is subdivided into dry and humid forest based on the proportion identified in the country in the GLC 2000 map.

**References**

FAO (2001) *Global Forest Resources Assessment 2000*. Forestry Paper 140. Food and Agriculture Organization of the United Nations, Rome.

FAO (2010) *Global Forest Resources Assessment 2010*. Forestry Paper 163. Food and Agriculture Organization of the United Nations, Rome.

Mayaux, P., Bartholomé, E., Fritz, S. & Belward, A. (2004) A new land‐cover map of Africa for the year 2000. *Journal of Biogeography*, **31,** 861–877.
